# Big Data Science on T Cell Receptor-mediated Immune Regulation

**DOI:** 10.31662/jmaj.2024-0304

**Published:** 2025-03-21

**Authors:** Kazuyoshi Ishigaki

**Affiliations:** 1 Department of Microbiology and Immunology, Keio University School of Medicine, Tokyo, Japan; 2 Keio University Human Biology-Microbiome-Quantum Research Center (WPI-Bio2Q), Tokyo, Japan; 3 Laboratory for Human Immunogenetics, Riken Center for Integrative Medical Sciences, Kanagawa, Japan

**Keywords:** T-cell receptor, thymic selection, autoimmune diseases, *HLA* polymorphisms, regulatory T cells

## Abstract

T cell receptors (TCRs) have a highly diverse sequence pattern resulting from the random recombination of gene components in the thymus. This diversity enables TCRs to distinguish between a wide range of self and non-self-antigens, thereby shaping the reactivity of the acquired immune system. Self-responsiveness arising from impaired TCR-based self-discrimination is a crucial trigger for the development of autoimmune diseases. The immunological importance of TCR research is evident, yet traditional experimental and analytical techniques have not fully captured the vast information contained within the TCR repertoire. However, recent advancements in massive parallel sequencing, efficient library preparation pipelines, single-cell experiment platforms, and genome engineering are poised to transform our understanding of TCR diversity, sparking interest in the field. These advancements have made it possible to "read through" the entire TCR repertoire and partially identify their cognate antigens. In parallel, methods for efficiently analyzing large datasets of comprehensive TCR sequences have also progressed. These innovations in experimental and analytical techniques are leading TCR research in new directions, such as using TCR as a real-time biomarker, exploring the link between TCR and T cell differentiation, and investigating TCR genetic regulation. This review will cover recent updates on big data science related to TCR-mediated immune regulation.

## Introduction

**Figure 1.**
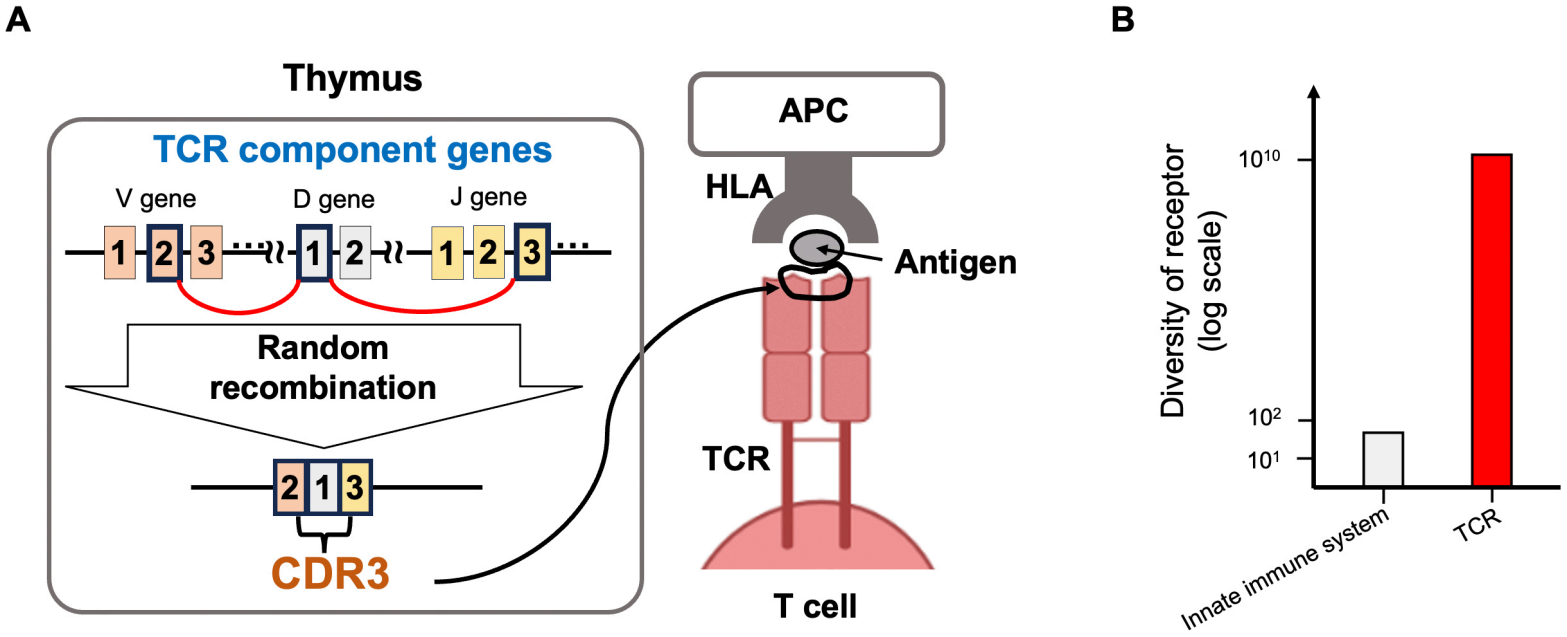
Basic information about T cell receptor. A. In the thymus, TCRs are generated through the random recombination of their component genes: V, D, and J genes. At their junctional region, several nucleotides are randomly added or deleted, resulting in significant diversity in this region. This junctional region is known as CDR3, which directly recognizes antigens presented on HLA molecules. B. The diversity of TCR receptors compared with innate immune system receptors. APC: antigen-presenting cell; CDR3: Complementarity-determining Region 3; TCR: T cell receptor.

T cells, the command center of the immune system, have T cell receptors (TCRs) on their surfaces. The TCR is the most important molecule for discriminating between self and non-self. TCRs exhibit a vast diversity of sequence patterns, with over 10^10^ potential combinations, due to the random genetic recombination of multiple component genes during T cell differentiation in the thymus (Figure 1). The joining region of these component genes is very short, averaging about 15 amino acids in length, and is known as the Complementarity-determining Region 3 (CDR3). During recombination, several nucleotides are added or deleted randomly, enhancing the sequence diversity. The CDR3 physically contacts antigenic epitopes and is the most critical site for antigen recognition ^(1)^. A major component of TCR diversity arises from TCR-CDR3 diversity. TCRs utilize this highly diverse sequence pattern to recognize a wide variety of corresponding antigens. Historically, researchers examined TCRs using spectrotyping ^(2)^, gel electrophoresis ^(3)^, and Sanger sequencing ^(4)^; however, these methods failed to capture the complete view of the TCR repertoire. In 2009, Robin et al. ^(5)^ published the first TCR repertoire study utilizing next-generation sequencing (NGS), which enabled a comprehensive evaluation of TCRs. Since then, we have observed many NGS-based TCR studies that have revolutionized our understanding of TCRs ^(6), (7), (8), (9), (10)^. For example, numerous studies have utilized TCR clonotypes as sequencing tags for T cells, illuminating the magnitude of clonal expansion ^(11)^, the size of the TCR repertoire in individual donors ^(12)^, the shared and distinct repertoires in different tissues of a donor ^(13)^, and the widespread presence of public clonotypes shared among various donors ^(6)^. In addition, many researchers have deposited TCR datasets in public domains, contributing to open science and enabling follow-up studies by other research groups. An important aspect of TCR repertoire analysis is that TCR sequence data contain rich information about T cell biology, serving as more than just simple molecular tags. In this review, we will introduce our recent studies on the genetic control of the TCR repertoire and the TCR features of regulatory T cells, and the latest research on the search for cognate antigenic epitopes recognized by TCRs.

## V2F Studies in Genetics

Before introducing the association between TCRs and *HLA* risk alleles for autoimmunity, we briefly provide background information on some basic concepts in human genetics. A genome-wide association study (GWAS) is a research method that detects associations between individual differences in genome sequences (polymorphisms) present at birth and human phenotypes. In non-genetic research, even when a strong association exists, it does not prove a causal relationship. However, because the phenotype cannot influence polymorphisms, GWAS is one of the few human studies capable of evaluating the causal biological mechanisms underlying the onset of human diseases. Over the past 20 years, large-scale GWAS have been conducted for autoimmune diseases. Typical examples include rheumatoid arthritis (RA) and systemic lupus erythematosus (SLE), in which GWAS has successfully identified risk polymorphisms in over 100 regions ^(14), (15), (16)^. Since GWAS findings suggest causal mechanisms of human diseases, they have the potential to advance our understanding of autoimmune disease pathogenesis, as well as to contribute to novel drug target discovery and personalized medicine.

**Figure 2.**
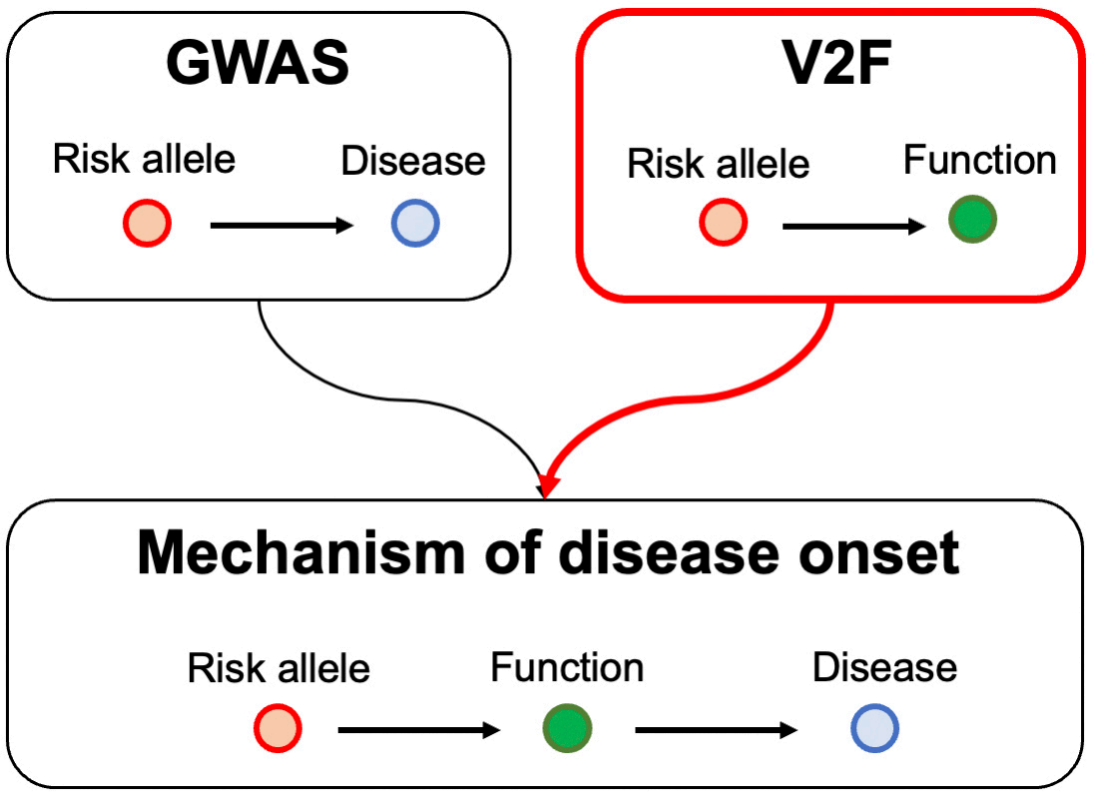
GWAS and V2F study. GWAS establishes the link between risk alleles and disease, while V2F connects risk alleles to biological function, such as TCR sequence patterns discussed in this manuscript. By integrating GWAS and V2F findings, we can hypothesize the causal mechanisms underlying disease onset. GWAS: genome-wide association study; V2F: variant-to-function.

However, the primary information obtained from GWAS is merely a collection of statistical data on polymorphisms across the entire genome. To extract meaningful biological insights from GWAS results, it is necessary to conduct extensive genetic research that links variants to their functions. This type of research is referred to as variant-to-function (V2F) research. By integrating the results of GWAS with those of V2F research, it becomes possible to infer the causal mechanisms underlying autoimmune diseases (Figure 2). With this in mind, V2F research is being actively pursued around the world.

**Figure 3.**
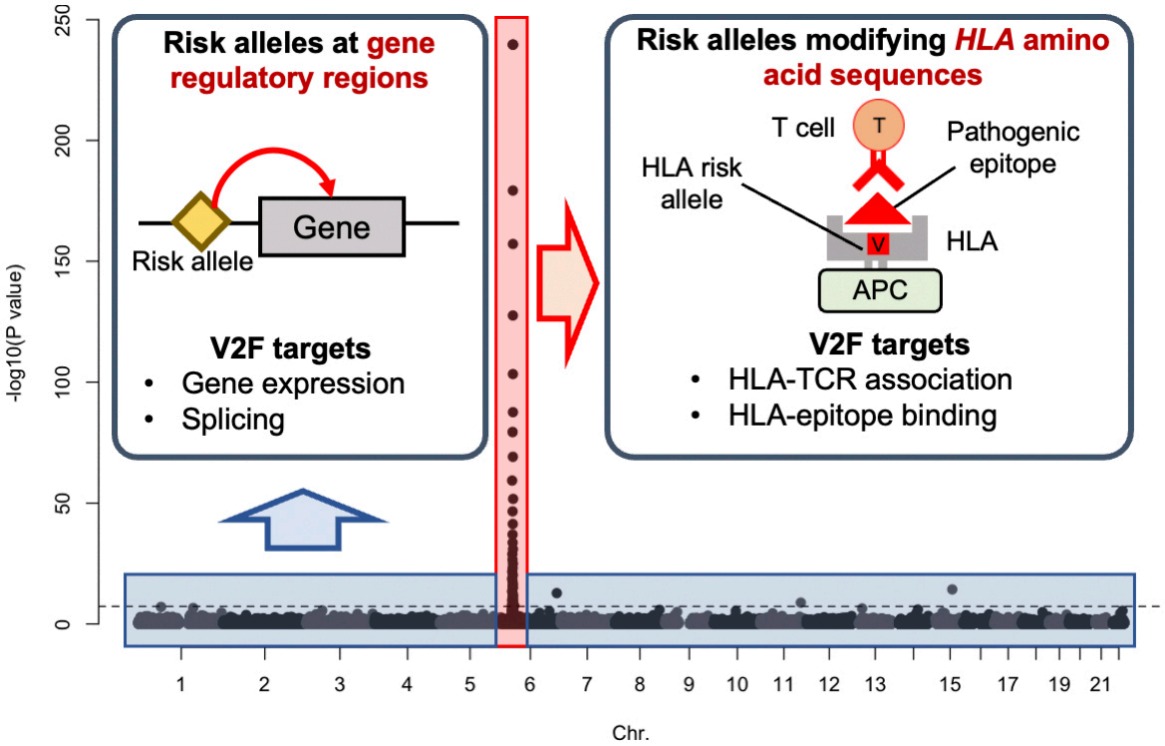
Rheumatoid arthritis risk allele distributions and functions. On the back, the results of the rheumatoid arthritis GWAS are presented. The X-axis indicates chromosomal position, while the Y-axis represents −log10(*P* value) of the GWAS, suggesting the strength of the association. Numerous but weak risk alleles located in the non-*HLA* regions are found in gene regulatory regions, such as enhancers, potentially affecting the expression of nearby genes. In contrast, a few very strong risk alleles in the *HLA* region modify HLA amino acid sequences, potentially influencing epitope binding efficiency and TCR thymic selection. APC: antigen-presenting cell; GWAS: genome-wide association study; TCR: T-cell receptor.

The most striking feature of GWAS for autoimmune diseases is the strong signal resulting from individual differences in the amino acid sequence of the *HLA* gene (amino acid polymorphism). In RA, about half of the genetic risk can be attributed to this polymorphism ^(17)^. In contrast, the other half of the genetic risk is distributed across various gene expression regulatory regions of the entire genome (Figure 3). Since the immunological functions of amino acid polymorphisms in the *HLA* gene and polymorphisms in gene expression regulatory regions are assumed to be completely different, it is necessary to conduct V2F research specific to each area. However, V2F research focusing on amino acid polymorphisms in *HLA* has not yet been sufficiently performed. To address the current limitations in genetics research, we have recently developed a novel analysis method to evaluate individual differences in TCRs and clarified that amino acid polymorphisms in *HLA* are strongly associated with individual differences in TCRs ^(18)^. In the next section, we will introduce the direction of new V2F research that focuses on the interaction between HLA molecules and TCRs.

## Associations between *HLA* Polymorphisms and T Cell Receptor Sequence Patterns

Autoimmune diseases are a group of disorders in which the immune system mistakenly attacks the body’s tissues. The exact cause of these diseases is unknown, but it is believed that abnormalities in the immune system’s ability to distinguish between self and non-self contribute to their onset. Recent large-scale genome analyses have revealed that the polymorphisms with the strongest effects on the risk of developing autoimmune diseases are located in the *HLA* gene region. In particular, it has been confirmed that the 13th and 71st amino acid polymorphisms in the *HLA-DRB1* gene significantly influence the risk of multiple autoimmune diseases, such as RA ^(17), (19), (20)^. However, it remains unclear how these risk polymorphisms in the *HLA* gene affect the immune system and contribute to abnormal self/non-self-discrimination.

In the development and differentiation of T cells in the thymus, undifferentiated T cells can only survive if their TCRs exhibit a weak affinity for self-antigens presented by HLA molecules. Conversely, they do not survive if they show a strong affinity. Thus, during the process of T cell selection in the thymus, polymorphisms in the *HLA* gene may influence the sequence patterns of TCRs. With this background, we hypothesized that risk polymorphisms in *HLA* genes (*HLA* risk polymorphisms) affect T cell selection in the thymus, leading to an increase in the number of autoreactive T cells and subsequently raising the risk of developing autoimmune diseases. To test our hypothesis, we conducted the following analyses.

If our hypothesis is correct, *HLA* risk polymorphisms should influence the frequency of TCRs with specific sequence patterns in the peripheral blood of healthy individuals. We aimed to analyze the relationship between *HLA* gene polymorphisms and TCR sequences using big data on TCR sequences collected from the peripheral blood of approximately 800 healthy individuals ^(6), (21)^. To accomplish this, we developed a new analysis algorithm that treats TCR sequence data as quantitative data and comprehensively evaluates its relationship with *HLA* gene polymorphisms ^(18)^. In this analysis, the strongest effect among all *HLA* gene polymorphisms was observed in the 13th amino acid polymorphism of the *HLA-DRB1* gene. Furthermore, when controlling for the effect of the 13th amino acid polymorphism of *HLA-DRB1*, the 71st amino acid polymorphism emerged as the next strongest factor. These results indicate that amino acid polymorphisms at the same site of the HLA molecule significantly impact both the risk of developing autoimmune diseases and the TCR sequence pattern.

Next, we assessed the specific effects of *HLA* risk polymorphisms on TCR sequence patterns in detail ^(18)^. Since multiple polymorphisms in the *HLA* gene region typically influence the risk of autoimmune diseases, we evaluated the cumulative effect of these polymorphisms rather than assessing each *HLA* risk polymorphism individually. This analysis revealed that the cumulative effects of *HLA* risk polymorphisms manifest different patterns for each disease. For example, *HLA* risk polymorphisms associated with RA and type 1 diabetes increase the number of acidic (negatively charged) amino acids in the TCR-CDR3 region, while those associated with celiac disease increase the number of hydrophobic amino acids. These analyses confirm that *HLA* risk polymorphisms affect the frequency of TCRs with specific sequence patterns in the peripheral blood of healthy individuals.

**Figure 4.**
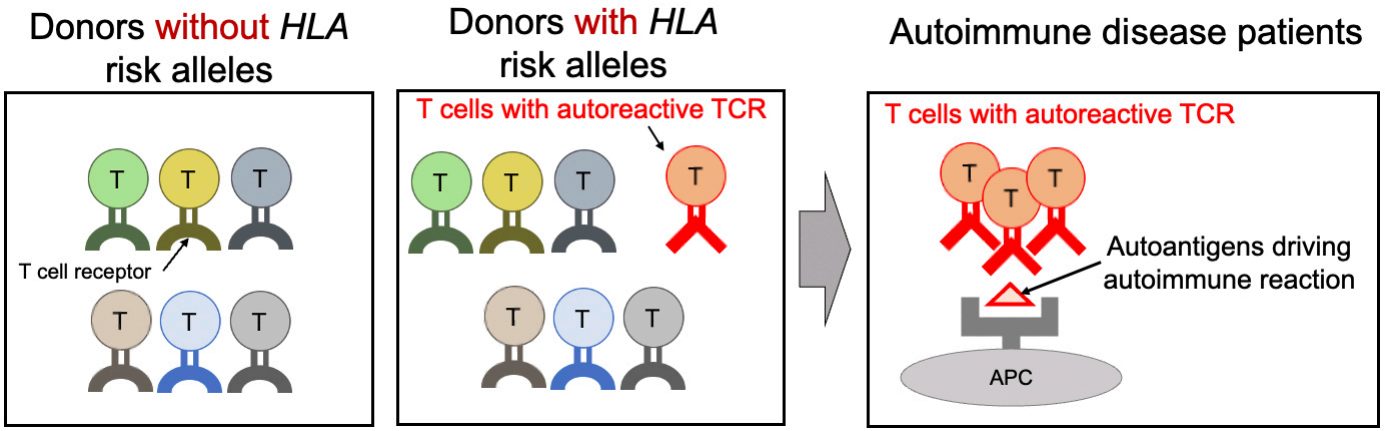
*HLA* risk alleles increase the frequency of autoreactive TCR. This illustration presents our hypothesis as discussed in the main text. Donors with *HLA* risk alleles have a higher prevalence of T cells with autoreactive TCRs in their peripheral blood compared to donors without *HLA* risk alleles, predisposing them to autoimmune reactions. In patients with autoimmune diseases, T cells possessing autoreactive TCRs undergo clonal expansion, driving the pathology associated with these diseases. APC, antigen-presenting cells. APC: antigen-presenting cell; TCR: T cell receptor.

Finally, we confirmed whether the TCR sequence patterns associated with *HLA* risk are involved in immune responses to autoantigens ^(18)^. To efficiently carry out this confirmation, we established a method for calculating an index (CDR3 risk score) that indicates the degree of accumulation of these sequence patterns in each TCR sequence. One of the characteristic pathological features of RA is an autoimmune response to citrullinated self-antigens. We collected T cells from the synovium of RA patients that react to various citrullinated self-antigens and calculated their CDR3 risk scores. Interestingly, we found that TCRs reacting with citrullinated autoantigens had higher CDR3 risk scores than other TCRs. These results support our hypothesis that the sequence patterns of TCRs modified by HLA risk polymorphisms promote immune responses to autoantigens (Figure 4).

## T Cell Receptor as a Real-time Biomarker

In the previous section, we discussed our observation of RA *HLA*-risk-associated TCR sequence patterns in the peripheral blood of healthy donor cohorts. These TCR patterns may reflect the abundance of pathogenic-epitope-responsive T cell populations. A reasonable follow-up expectation is that we can utilize peripheral blood TCR repertoire information as a biomarker to predict the risk of developing RA and other autoimmune diseases. Supporting this expectation, a previous study reported substantial differences in peripheral blood TCR repertoire patterns between RA patients and healthy donors ^(7)^. Additionally, TCRs reflect immune memory from past exposure to various pathogens. For example, previous studies found that donors who had been infected with cytomegalovirus shared public clonotypes (identical TCR-CDR3 sequences) in their peripheral blood TCR repertoire data, demonstrating their utility as real-time biomarkers of immune status ^(6)^. The research group also reported similar observations for coronavirus disease 2019 infection ^(22)^.

## T Cell Receptor Sequence Patterns and T Cell Fate

Regulatory T cells are a subset of helper T cells that play a crucial role in restraining the immune response. Abnormalities in regulatory T cells are implicated in many diseases. When helper T cells differentiate in the thymus, they are exposed to various self-antigens. It is known that when the TCR exhibits a high affinity for self-antigens, some of these helper T cells differentiate into regulatory T cells. Through this mechanism, regulatory T cells recognize self-antigens and suppress immune responses to them, thereby preventing the onset of autoimmune diseases. With this in mind, we hypothesized that specific TCR sequence patterns increase reactivity to self-antigens and are involved in the differentiation of regulatory T cells in the thymus. To test our hypothesis, we conducted the following analysis.

We analyzed a large dataset (approximately 60 million sequences) of TCR sequences collected from 65 individuals to evaluate the characteristics of TCRs in regulatory T cells compared to those in other helper T cells ^(23)^. First, to understand the composition of the dataset, we measured the proportion of amino acids present in the CDR3 region of the TCR. We found that the frequency of amino acids such as leucine and phenylalanine was significantly higher in regulatory T cells. Interestingly, we noticed that the amino acids frequently used in regulatory T cells share the common property of being hydrophobic. This finding suggests that the hydrophobicity of the CDR3 region may enhance the recognition of self-antigens.

**Figure 5.**
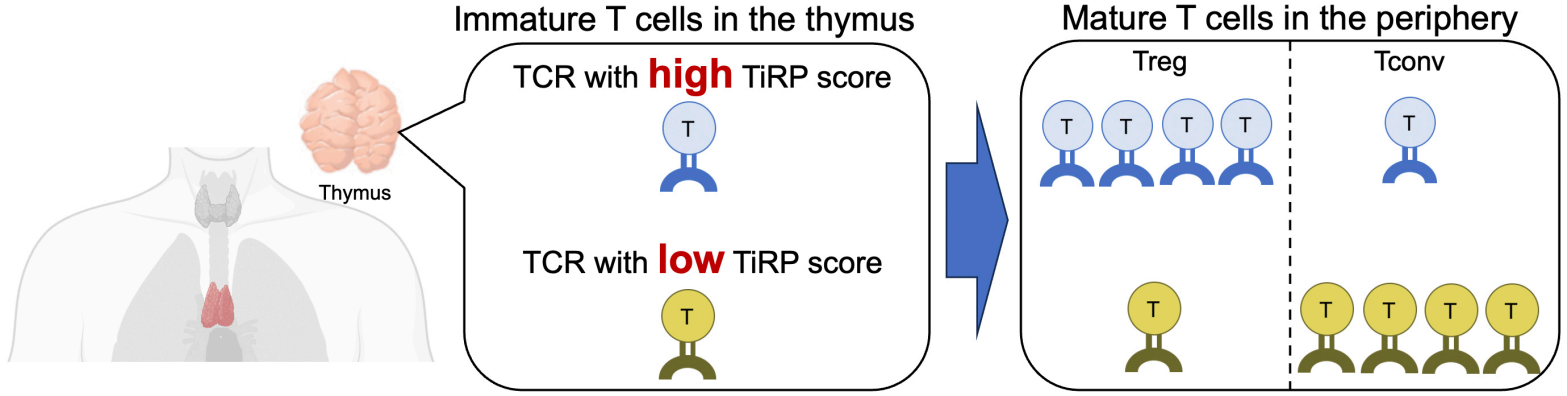
TCR amino acid features define T cell fate. This illustration presents our hypothesis, as discussed in the main text. When a T cell has a TCR enriched with specific amino acid features (i.e., high TiRP score), that T cell is more likely to differentiate into Tregs rather than Tconvs. The illustration of the thymus was created using BioRender. Tconv: conventional T cell; TCR: T cell receptor; TiRP: TCR-intrinsic regulatory potential; Treg: regulatory T cell.

We extracted all the characteristics of each amino acid in the CDR3, V, and J genes (a total of 606 types) from the TCR sequences and exhaustively searched for the characteristics of TCR sequences in regulatory T cells by applying a generalized linear mixed model that we devised ^(23)^. As a result, we identified a total of 208 features of TCR sequences that are important for the differentiation of regulatory T cells. Furthermore, we established a method for calculating a score (TCR-intrinsic regulatory potential [TiRP]) that quantifies the extent to which each TCR sequence contains these characteristics. The TiRP score serves as an indicator of the “regulatory T cell-like nature” of the TCR, and helper T cells with TCRs that have high TiRP scores are more likely to differentiate into regulatory T cells than those with low scores (Figure 5). When we evaluated the utility of the TiRP score using an independent dataset, we demonstrated that the TiRP score was strongly associated with the likelihood of differentiation into regulatory T cells. For instance, the ratio of regulatory T cells to other helper T cells was approximately 1:3 in the highest percentile of the TiRP score, compared to approximately 1:12 in the lowest percentile.

It is known that some regulatory T cells can change their phenotype in peripheral tissues and transform into other helper T cells, demonstrating the plasticity of regulatory T cells. Most findings on the plasticity of regulatory T cells have been derived from experiments using model mice, and detailed discussions concerning humans have not been fully conducted. Therefore, we applied the TiRP score to human TCR data to investigate the mechanisms underlying regulatory T-cell plasticity. T cells that express both regulatory T cell and other helper T cell phenotypes (mixed helper T cells) can be considered a population of helper T cells that exhibit plasticity ^(23)^. We found that the TiRP scores of these mixed helper T cells were intermediate between those of regulatory T cells and other helper T cells. This finding suggests that the sequence patterns of TCRs influence the plasticity of regulatory T cells in the human immune system.

## Cognate Antigen Search of a Given T Cell Receptor

Finally, we introduce several research activities aiming to identify cognate antigens of a given TCR. Antigen-specific immune responses play a key role in the pathology of many diseases. For example, autoimmune diseases are intractable diseases in which the immune system attacks self-antigens, mistaking them for non-self, and tissue damage occurs. The antigen-specific immune reaction is also responsible for eliminating abnormal antigens that are specifically expressed in cancers. However, in many cases, it is not clear what antigens the immune system is reacting to before the onset of diseases. Due to this limitation, the majority of existing drugs are designed to suppress the immune response in antigen non-specific manner. As a result, there is always a risk of side effects with existing drugs, and the therapeutic response is also limited. Therefore, there is a need to develop antigen-specific immunotherapy. If we were able to predict the cognate antigens from the TCR sequence information, the antigen that causes the essential pathological condition will become clear, and this will provide the basic information for the development of antigen-specific immunotherapy.

As discussed above, massive TCR databases are widely available, and TCR evaluation will become a standard part of assessing patients’ immune status. However, a key challenge in identifying cognate antigens is the lack of an experimental system enabling high-throughput screening. Kula et al. ^(24)^ established one such system known as T-Scan, in which a lentiviral library is used to infect a cell line, creating a population of “artificial antigen-presenting cells” that express various antigens. These artificial antigen-presenting cells contain a reporting system to detect granzymes released by T cells when an antigen-specific immune response occurs. The target T cell population is then co-cultured with the artificial antigen-presenting cells, and those that have generated an antigen-specific immune response are identified using the granzyme reporting system. By evaluating these cells through sequencing, it is possible to identify the sequence of the infecting lentivirus and the corresponding antigen.

## Conclusions

The widespread use of next-generation sequencers has led to the accumulation of vast amounts of TCR data. The majority of TCR datasets presented in this article were obtained from public databases of TCR sequences that are available for free download to researchers in academic institutions. NGS-based TCR research is still in its infancy, and many scientifically important questions remain unanswered. We expect that TCR research will continue to develop rapidly in the future, leading to significant findings in the field of immunology.

One of the most important unanswered questions is the physicochemical rules governing how TCR recognizes specific antigens. Decoding this mystery is essential for understanding and controlling our health. Thus, unraveling the rules behind TCR antigen specificity has been a central objective in immunology and a longstanding goal for many scientists. Although NGS-based TCR research helps address this issue, it is far from sufficient. We need to understand other aspects of TCR, such as its structure, and to do so, more experimental investigations on TCR are needed. The current bottleneck is the throughput of such experiments, which must be capable of coping with the vast diversity of TCRs.

## Article Information

This article is based on the study, which received the Medical Research Encouragement Prize of The Japan Medical Association in 2023.

### Conflicts of Interest

None

### Author Contributions

KI wrote the manuscript and made figures.
